# Oriental reed warblers retain strong egg recognition abilities during the nestling stage

**DOI:** 10.1002/ece3.11063

**Published:** 2024-02-20

**Authors:** Laikun Ma, Wei Liu, Peng Pan, Jianhua Hou, Wei Liang

**Affiliations:** ^1^ School of Life Sciences Hebei University Baoding China; ^2^ Department of Biology and Food Science Hebei Normal University for Nationalities Chengde China; ^3^ Ministry of Education Key Laboratory for Ecology of Tropical Islands, Key Laboratory of Tropical Animal and Plant Ecology of Hainan Province, College of Life Sciences Hainan Normal University Haikou China

**Keywords:** brood parasitism, egg mimicry, egg recognition, egg rejection, the crypsis hypothesis

## Abstract

Egg recognition and rejection are the most common and effective anti‐parasitic strategies against avian brood parasitism in terms of maintaining stability over time and plasticity in response to environmental cues. Conversely, parasites have evolved multiple counter‐adaptations to the anti‐parasitic defenses of hosts. Among them, the crypsis hypothesis suggests that eggs that appear more cryptic in color and are closely matched to the environment are helping to counter the egg recognition strategy of the host. In this study, we investigated whether the egg recognition ability of Oriental reed warblers (*Acrocephalus orientalis*), a common host of common cuckoos (*Cuculus canorus*), changed during different reproductive stages by using model egg experiments. The effect of the crypsis hypothesis on the egg recognition ability of the hosts was also investigated by controlling the color contrast between the inside of the experimental nests and the model eggs. The results showed that the Oriental reed warbler retained strong egg recognition abilities, which were similar to the incubation stage (GLMMs: *F*_1,27_ = 0.424, *p* = .521), even after entering the nestling stage and preferentially rejected model eggs with distinct contrasting colors (binomial test: Fisher's exact, *p* = .016). These results are consistent with the crypsis hypothesis. The present study suggests that the host retains a strong egg recognition ability even during the nestling stage and that cryptic‐colored eggs that are closely matched with the breeding nest environment help counter the host's egg recognition abilities and increase the chances of successful parasitism by cuckoos. However, the effectiveness of cryptic egg may be weaker than mimic egg in countering egg recognition and rejection by hosts with open‐cup nests.

## Graphical abstract

Oriental reed warblers preferentially rejected model eggs with distinct contrasting colors supporting the crypsis hypothesis.

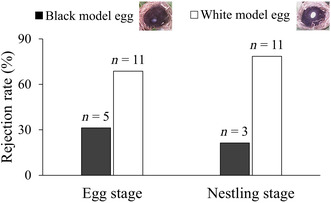

## 1 INTRODUCTION

Successful nest parasitism in exclusively parasitic birds can impose huge reproductive costs on the host, thus requiring hosts to evolve appropriate anti‐parasitic strategies (Lyu & Liang, 2021; Soler, 2014). Recognition and rejection of parasitic eggs are the most common and effective anti‐parasitic strategies of hosts and an important indication of host adaptation to nest parasitism (Davies & Brooke, 1989; Ma et al., 2022; Ma & Liang, 2021; Moksnes et al., 1991; Soler et al., 2017; Yang et al., 2020, 2021). Some hosts can acquire egg recognition rapidly under high parasitic pressure (e.g., Nakamura, 1990; Soler et al., 1994) and be maintained for a considerable period of time and be a rapid expression and response to the stimuli of alien egg, even after escape from parasitic stress or abandonment by cuckoos (Honza et al., 2004; Peer et al., 2007, 2011; Rothstein, 2001; Yang, Li, et al., 2014; Yang, Liu, et al., 2014; Yi et al., 2020). There are also some hosts that show greater flexibility in egg recognition abilities with rapid release or decrease in the absence of parasitism (Soler et al., 2012; Stokke et al., 2008; Yang, Wang, et al., 2015).

Different geographic populations of the same host species are subject to different parasitism risks and selection pressures and thus show significantly different egg recognition abilities (Liang et al., 2016; Lindholm & Thomas, 2000; Moskát et al., 2002). For example, Reed warbler (*Acrocephalus scirpaceus*), one of the most commonly used hosts by Common cuckoos (*Cuculus canorus*) in Europe, shows a significantly lower ability of egg rejection in population with no parasitism compared to a regularly parasitized population (Lindholm & Thomas, 2000). Differential egg recognition abilities can also be seen among hosts with different parasitism rates within the same region (Edvardsen et al., 2001; Kleven et al., 2004; Yang et al., 2021; Yang, Su, et al., 2015). On the contrary, the egg recognition abilities of the host also exhibit plasticity on a temporal scale, depending on the parasitism risk (Brooke et al., 1998; Nakamura, 1990; Thorogood & Davies, 2013). Hosts are capable of judging parasitism risk based on the presence of parasites within the same breeding season, adjusting their egg recognition and egg rejection behaviors, and fine‐tuning plasticity due to the inconsistencies in breeding periods between the parasite and host (Zhang et al., 2021). In addition, hosts also exhibit differential egg recognition during different reproductive stages, such as egg laying, incubation, and nestling, depending on the parasitism risk they are exposed to (Moskát, 2005; but see Davies & Brooke, 1989). Previous studies have focused on the egg‐laying and incubation stages. There are few studies on the egg recognition abilities of the host during the nestling period (but see Moskát, 2005). Additionally, a complex nest environment like the chicks accidentally push the egg out of the nest may result in low accuracy in the nestling stage.

Since some hosts have effective egg recognition abilities, parasites continue to optimize their parasitism strategies, such as egg mimicry. For example, Common cuckoos can mimic the egg patterns of some of its common hosts, making it difficult to distinguish between the host eggs and parasitic eggs (Li et al., 2016; Ma et al., 2022; Moksnes et al., 2013; Moksnes & Røskaft, 1995). How closely the parasite's egg mimics host eggs has been shown to reduce host rejection of parasitized eggs (e.g., Antonov et al., 2006, 2007; Li et al., 2016). In addition, in contrast to egg mimicry, some parasites of hosts with darker parasitic nest environments lay parasitic eggs of darker colors resulting in less visible in the nest, which is a potential alternate evolutionary trajectory possibly in response to egg recognition and rejection by the host, in support of the crypsis hypothesis (Brooker et al., 1990; Harrison, 1968; Langmore et al., 2009). Studies have found that cryptic‐colored eggs could play a role in host recognition and rejection (Antonov et al., 2011; Wang, He, et al., 2021; Yang et al., 2023), avoiding egg‐removing behavior by other parasites in a nest suffering multiple parasitism (Gloag et al., 2014; Thorogood et al., 2017; Wang, Zhang, et al., 2021), and reducing predation risk (Mason & Rothstein, 1987). However, many studies have also found that hosts did not alter egg recognition and rejection responses via increasing nest light intensity or shifting in egg‐nest contrasts experimentally (Aidala et al., 2015; Hauber et al., 2015; Medina & Langmore, 2019). Therefore, compared to the extensive research on egg mimicry, there are fewer studies on the cryptic egg hypothesis, especially in open‐nesting species, and the results are inconsistent.

The Oriental reed warbler (*Acrocephalus orientalis*), a favorite host of Common cuckoos in China, is at a high stage of coevolution with cuckoos which the cuckoo has formed a highly mimetic parasitic egg including the size and coloration in the local population (Li et al., 2016; Ma et al., 2022; Ma, Yang, Liu, et al., 2018; Yang, Li, et al., 2014). The Oriental reed warblers can recognize and reject nearly 100% of the non‐mimetic eggs and some highly mimetic cuckoo eggs during the egg stage (Li et al., 2016; Ma et al., 2022; Ma & Liang, 2021), but whether such high egg rejection retains during the nestling stage is unclear. Concurrently, parasitism rates differed significantly between the Oriental reed warbler population studied by Wang, He, et al. (2021) and the population in this study. Both populations differed in parasitism risk (14.8 vs. 34.3%, Ma, Yang, Liu, et al., 2018; Yang, Li, et al., 2014), and conspecific hosts exhibited geographic variation and different behavioral responses to similar alarm sound playback between the two populations (Wang et al., 2020; Wang & Yang, 2020; Yu et al., 2019). Therefore, this study can also further test the applicability of the crypsis hypothesis and study egg recognition ability during the nestling stage drawing on the method of Wang, He, et al. (2021) in different geographic populations with low risk of parasitism. We predicted that (1) If egg rejection behavior occurs, the warblers would preferentially reject more distinguishable foreign eggs because crypsis egg colors may reduce the probability of detection according to the crypsis hypothesis. (2) The egg rejection ability of warblers will decrease or disappear during the nestling stage due to the lack of parasitic pressure.

## 2 MATERIALS AND METHODS

### 2.1 Study area and species

The study area was located in Yongnianwa National Wetland Park of Hebei Province (36°40′–36°41′ N, 114°41″–114°45′ E). The main plants are reeds (*Phragmites australis*) and cattails (*Typha latifolia*). The Yongnianwa Wetland has a well‐developed water system and numerous tributaries at an altitude of only 40.3 m. The average annual rainfall is 527.8 mm, mostly in summer, and the average annual temperature is 12.9°C (Ma, Yang, Liu, et al., 2018). The Oriental reed warbler is one of the main hosts of Common cuckoos, which is the most common obligate interspecific brood parasitic bird breeding in Europe and Asia. The coevolution between the two species has been proposed to have reached a high level (Li et al., 2016; Ma et al., 2022). The probability of the Oriental reed warbler being parasitized by cuckoos in the Yongnianwa area is significantly lower than in the population studied by Wang, He, et al. (2021), enabling this study to make a good comparison under new contexts (14.8 vs. 34.3%, Ma, Yang, Liu, et al., 2018; Yang, Li, et al., 2014).

### 2.2 Field experiments

Fieldwork was conducted during late June and late July 2016–2017 in the late breeding season. Breeding nests in the study area were systematically searched for, and the experiments were conducted on nests entering the incubation and nestling stages ignoring the incubation period and nestling age due to insufficient nests in the late breeding season. Based on the method of Wang, He, et al. (2021) and Trnka et al. (2012), a particular direction from the target nests of Oriental reed warblers was randomly selected, and two old nests (the nests were collected after breeding was completed previously) were placed 0.5 m from the target nest. According to Wang, He, et al., 2021 and our own field observations, the warblers would visit and check experimental nests quickly and then reject the model eggs in some trials, perhaps the alien nests may have encroached on their breeding territory. The two old nests were filled with black wool to simulate a dark environment. One black model egg made of black synthetic clay was placed inside one old nest to simulate a cryptic egg color (21.42 ± 0.30 × 15.37 ± 0.25 mm, egg mass: 4.17 ± 0.02 g, mean ± SD; *n* = 15), and one white model egg made of white synthetic clay was placed inside the other old nest to simulate a bright egg color (21.34 ± 0.26 × 15.48 ± 0.27 mm, egg mass: 4.16 ± 0.02 g, mean ± SD; *n* = 15). We did not measure the contrasting color differences quantitatively between nest with black interiors and model eggs of different colors in the field. However, a similar treatment according to Wang, He, et al. (2021) showed the color contrast of black model eggs in a black environment was significantly lower than that of white model eggs. The responses of the Oriental reed warbler to the model eggs in the two nests were recorded by rejection or acceptance. If the model eggs in either nest showed distinct pecking marks or disappeared, it was considered a rejection, and the experimental trial was completed. If the host checked the nest but the model egg remained undamaged until the sixth day, it was deemed to be accepted. If there were rejections in both nests, the order of rejection would be determined using video‐assisted recordings with a mini‐camera (JWD DV‐58, Jingwah Electronics Co., Ltd., Shenzhen, China). Each nest was performed experiment only once either in the egg stage or nestling stage, respectively.

### 2.3 Statistical analysis

Statistical analyses were conducted using IBM SPSS 26.0 for Windows and generalized linear mixed models (GLMMs) to predict the effects on whether eggs were removed (rejected vs. accepted) and the color preference for removed eggs (black model egg vs. white model egg) at different breeding stages of the Oriental reed warbler. The dependent variables of the first model were whether eggs were removed (rejected vs. accepted) and the second model was the color preference for removed eggs (black vs. white model egg). The year, date, clutch size, breeding stage and breeding progress (incubation days or nestling age) were fixed effects. The number of each experimental nest was a random effect. Fisher's exact test was used for comparison between different probabilities like the egg rejection rate under different situations. Binomial test (Fisher's exact) tested the rejection preference of the Oriental reed warbler for model eggs of both colors. The data from the incubation and nestling stages were combined for further comparison when the breeding stage had no influence on egg recognition and rejection preferences. All tests were two‐tailed, with a significance level of *p* < .05, and data were represented in the form of mean ± standard deviation (Mean ± SD).

## 3 RESULTS

**FIGURE 1:**
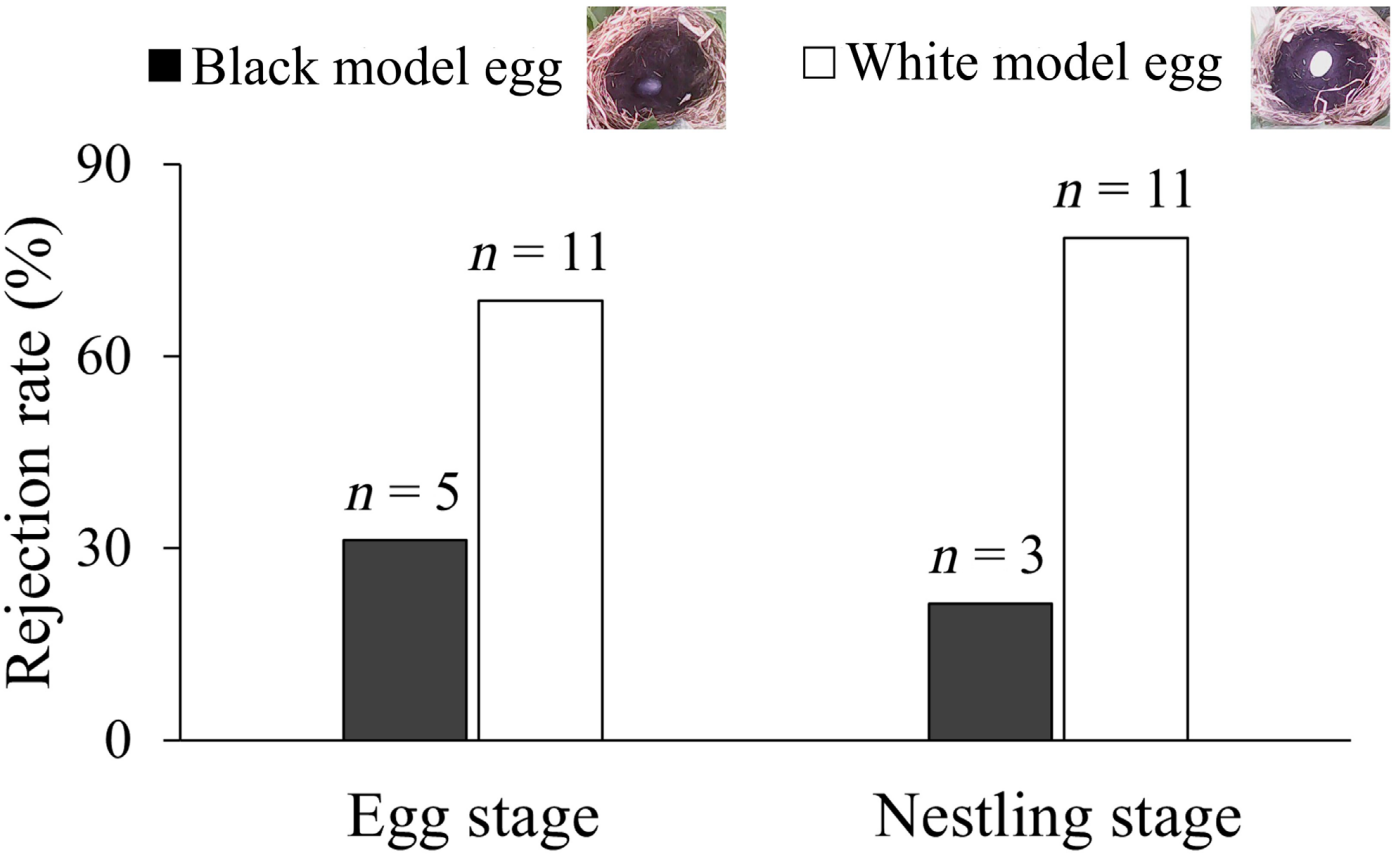
The rejection rate of Oriental reed warblers of white and black model eggs in dark nest environments.

A total of 38 trials were tested across both years. For the 21 trials with the rejection rate of 76% in the incubation stage (7.24 ± 2.05, *N* = 17 for average incubation days), 31% of rejection trials were recorded preferentially in nests with black model eggs, and 69% occurred in the nests with white model eggs. For the 17 trials with the rejection rate of 82% in the nestling stage (4.50 ± 2.83, *N* = 16 for average nestling age), 21% of rejection trials were recorded preferentially in nests with black model eggs, and 79% occurred in the nests with white model eggs were rejected (Figure 1 and Video S1 and S2). The egg rejection rate to the neighbor nest in this study was lower than own nest in previous studies for both incubation and nestling periods (Fisher's exact test: *p*_1_ = .005; *p*_2_ = .042).

GLMMs revealed no difference in egg recognition and rejection between incubation and nestling stages (breeding stage: *F*_1,27_ = 0.424, *p* = .521) (Table 1). There was no difference in the egg recognition and rejection preferences of model eggs of different colors between the incubation and nestling stages (breeding stage: *F*_1,26_ = 0.330, *p* = .572). Therefore, the data from the incubation and nestling stages were combined, and it was found that the Oriental reed warbler preferentially rejected white model eggs that contrasted with the nest environment (binomial test: *p* = .016) (Figure 1).

**TABLE 1:** Generalized linear mixed models (GLMMs) used to predict the response toward the model egg and rejection preference for the egg color with black and white in different breeding stages.

	*F*	df 1	df 2	*p*
Model for egg rejection				
Year	0.231	1	27	.635
Date	0.415	1	27	.525
Breeding stage	0.424	1	27	.521
Clutch size	0. 054	1	27	.817
Breeding progress	0.551	1	27	.464
Model for rejection preference				
Year	1.162	1	21	.293
Date	0.625	1	21	.438
Breeding stage	0.330	1	21	.572
Clutch size	0.132	1	21	.720
Breeding progress	0.118	1	21	.734

## 4 DISCUSSION

In this study, we found that the Oriental reed warbler retained strong egg recognition and rejection abilities during the nestling period that were similar to the incubation stage. Additionally, the Oriental reed warbler preferred to reject model eggs with distinct contrasting colors to the nest environment, while cryptic‐colored eggs similar to the nest environment had an advantage in countering egg recognition by the host, which supported the crypsis hypothesis.

Contrary to our second prediction, this study found that the Oriental reed warbler exhibited high rejection ability toward model eggs in neighboring nests during the nestling period (*r* = 82.35%, *n* = 17), which was similar to the incubation stage. One possible explanation is infanticide, in which parent birds destroy conspecific nestlings or eggs in nearby nests (Hansson et al., 1997; Trnka et al., 2010). Studies on Great Reed Warblers (*Acrocephalus arundinaceus*) have found that female parent birds engage in nest destruction of neighboring conspecifics, and the proportion is higher in polygynous individuals due to competition for resources from males (Trnka et al., 2010). The Oriental Reed Warblers in this study are closely related to the Great Reed Warbler with different behaviors (Trnka et al., 2023), but the mating system has not been detected in this population for the Oriental Reed Warblers. However, it is unlikely that conspecific destruction is responsible for such a high rate of egg rejection (82.35% in our study vs the highest rejection rate of 37% in a previous study). Based on the recording videos for egg rejection, the Oriental Reed Warbler quickly grasps and throws away the model egg within neighboring nests, which is more like egg ejection than egg destruction. In addition, the Oriental Reed Warbler population in this study has a high population density, with the nearest conspecific natural nests being only about 2 m away, and there is a high proportion of neighbor‐assistance behavior in nest defense in this population (Ma, Yang, & Liang, 2018; Ma, Yang, Liu, et al., 2018). Tolerance of neighbor presence may be more advantageous in countering parasitism due to more individuals could effectively resist the cuckoos among the Oriental Reed Warbler populations with cooperative defense (Wang et al., 2023).

In contrast, we believe that the rejection of model egg in this study is more likely to be a continuation of egg recognition behavior, where the removal of non‐conspecific model eggs from neighboring nests is an expression of egg recognition stimuli. A similar study on the Oriental Reed Warbler in another population during the egg stage also found that they also rapidly reject model eggs in neighboring nests (Wang, Zhang, et al., 2021). Previous studies have suggested that hosts can fine‐tune their egg recognition abilities in response to external parasitism risk (Lindholm & Thomas, 2000; Zhang et al., 2021). Moskát (2005) found that hosts retained only low egg recognition abilities during the nestling stage without parasitism risk, consistent with the dynamic risk assessment hypothesis (Kleindorfer et al., 2005; but see Peer et al., 2007).

Possible reasons for the difference may be the study by Moskát (2005) directly added model eggs to the nest with nestlings, possibly leading to the model eggs being accidentally pushed out of the nest by the nestlings and more likely to be underneath the nestlings. This could result in the parents being unable to examine the model eggs, possibly causing a low rejection rate. Parents would devote more time and effort to rear nestling during the nestling period, and lower nest inspection may affect the rate of egg recognition and rejection (e.g., Davies & Brooke, 1989). The Oriental reed warbler population in this region rarely made egg recognition errors in our study or previous egg recognition studies (Ma et al., 2022; Ma & Liang, 2021). This suggests that the cost of maintaining egg recognition abilities in this region is not high, and thus the short‐term loss of parasitic pressure during the nestling stage was insufficient to create strong selection pressure (e.g., Rothstein, 2001; Yang, Liu, et al., 2014). Previous studies have also found that many host species (e.g., the redbilled leiothrix *Leiothrix lutea*, the gray catbird *Dumetella carolinensis*) escaped from parasitism also exhibit strong egg recognition ability, considering that egg recognition and rejection are conditional behavioral responses to particular stimuli such as parasitic eggs and may be close to neutral in adaptive value in the absence of such stimuli, with the maintenance of these traits requiring minimal costs (e.g., Peer et al., 2007; Rothstein, 2001; Yang, Liu, et al., 2014).

This study also found that the egg rejection rate was lower than in previous studies (for both incubation and nestling periods Fisher's exact test: *p*_1_ = .005; *p*_2_ = .042) (Ma & Liang, 2021). This may be due to the following reasons: first, it may be that a model egg was added into the neighboring nests for this study, and removal of model eggs from neighboring nests was an expression of particular egg recognition stimuli which resulted in assisted defense behavior aimed to reduce the whole risk of parasitism. However, the frequency of parental inspection may be low for neighboring nest, which affects its egg rejection rate (Davies & Brooke, 1989). Similar assistance behavior has been found in a high proportion of nest defense for the Oriental reed warbler in different populations (Li et al., 2016; Ma, Yang, & Liang, 2018; Ma, Yang, Liu, et al., 2018; Wang et al., 2023). Second, only one egg was placed in the experimental nest in this study, similar to in the early egg‐laying period. However, previous studies have found reduced host egg recognition and a high rate of nest abandonment during the early egg‐laying period (Moskát, 2005), which could not be detected in this study. Wang, He, et al. (2021) placed four model eggs in a different population, and no reduced rejection rate was observed, which indirectly supports this theory. Finally, this experiment was conducted mainly in the middle and late part of the breeding season, where there were some first‐time breeding individuals. Egg recognition results from a combination of genetics and learning, with lower egg rejection rates among first‐time breeders (Lotem et al., 1992; Molina‐Morales et al., 2014; but see Amundsen et al., 2002).

The experimental nests used in this study contained two egg types with distinct contrasting colors placed in a dark nest environment. In line with our first prediction, the rejection rate of the white eggs with distinct contrasting colors was significantly higher than that of the black model eggs, consistent with the crypsis hypothesis (Harrison, 1968; Langmore et al., 2009). In general, there are two strategies to counter host egg recognition including egg mimicry and egg crypsis (Brooker et al., 1990; Harrison, 1968; Mason & Rothstein, 1987). The former is widely recognized as an effective strategy evolved to counteract the host's egg recognition ability and the interaction process proceeds and constitutes escalating coevolution (Soler, 2014). The crypsis hypothesis is used to explain exceptional cases where non‐mimetic parasitic egg would survive in host nests because of dim coloration which is cryptic enough to avoid detection by the host (Antonov et al., 2011; Wang, He, et al., 2021; Wang, Zhang, et al., 2021; Yang et al., 2023), secondary parasite (Gloag et al., 2014; Thorogood et al., 2017; Wang et al., 2020) or predator (Mason & Rothstein, 1987). Previous studies have found that some parasites lay dark‐colored parasitic eggs and mostly use hosts with darker nesting environments, presumably using darker nesting environments to hide darker parasitic eggs as a response to host egg recognition and rejection, in line with the crypsis hypothesis (Langmore et al., 2009). However, subsequent studies have found that dark‐colored parasitic eggs are more likely to be a response to the pecking of eggs by other parasites in multiple parasitism prior to egg laying rather than recognition pressure from the host (Gloag et al., 2014; Thorogood et al., 2017). Furthermore, some studies have found that increasing nest illumination or egg‐nest contrast did not alter host egg recognition and rejection ability (Aidala et al., 2015; Medina & Langmore, 2019; but see Yang et al., 2022), which is inconsistent with the cryptic egg hypothesis. However, research on Green‐backed Tits (*Parus monticolus*) has found that its egg discrimination disappeared when nest luminance was reduced to a minimum of 0.35 ± 0.15 lux which represents total darkness for humans (Yang et al., 2022), and further studies have shown that both the egg darkness and egg‐nest similarity affect egg recognition, and the former plays a more influential role, supporting the cryptic egg hypothesis (Yang et al., 2023). Wang, He, et al. (2021) and Wang, Zhang, et al. (2021) used different combinations of nest environments and model eggs of contrasting colors within well‐lit open‐cup nest and found that the common cuckoo and the Oriental reed warbler, preferentially detect and reject model eggs that differ significantly from the nest environment, supporting the crypsis hypothesis. Similar results in this study suggest that geographic populations subject to different parasitism risks are similar in their rejection rates of experimental eggs, as well as in the perception and selection behavior of experimental eggs of different contrasting colors (Ma & Liang, 2021; Wang, He, et al., 2021; Wang, Zhang, et al., 2021). Additionally, some black model eggs were also rejected by Oriental Reed Warblers in this study, and the rejection rate was significantly higher than toward real cuckoo mimic eggs shown by Li et al. (2016), indicating that the effectiveness of cryptic egg is lower than mimic egg in countering egg recognition and rejection by hosts with open‐cup nests.

In summary, the present study found that the Oriental reed warbler similarly retained strong egg recognition abilities during the nestling stage by using paired experiments of egg recognition with distinct contrasting colors during the incubation and nestling stages. Results showed that the Oriental reed warbler ignored black model eggs with less distinctly contrasting colors more often and that parasitic eggs with cryptic colors helped to counter host egg recognition, further validating and supporting the crypsis hypothesis. However, the effectiveness of cryptic egg may be weaker than mimic egg in countering egg recognition and rejection by hosts with open‐cup nests.

## AUTHOR CONTRIBUTIONS

**Laikun Ma:** Formal analysis (lead); funding acquisition (equal); investigation (lead); methodology (lead); writing – original draft (lead). **Wei Liu:** Software (equal); visualization (equal). **Peng Pan:** Software (equal); visualization (equal). **Jianhua Hou:** Resources (equal); supervision (equal); writing – review and editing (equal). **Wei Liang:** Conceptualization (lead); funding acquisition (equal); supervision (equal); writing – review and editing (equal).

## FUNDING INFORMATION

This work was supported by the National Natural Science Foundation of China (Nos. 32101242 to LM, 31970427 and 32270526 to WL).

## CONFLICT OF INTEREST STATEMENT

The authors declare that they have no competing interests.

## Supporting information


Table S1.

Video S1.

Video S2.

## Data Availability

The data that supports the findings of this study are provided in Table S1, Video S1 and S2.
